# Evaluation of the Efficacy and Safety of a Panthenol‐Enriched Mask for Skin Barrier Recovery After Facial Laser Treatment: Results of a Double‐Blind Randomized Controlled Study

**DOI:** 10.1111/jocd.70223

**Published:** 2025-07-04

**Authors:** Meiyan Gao, Ni Gao, Li Wang, Ting Yao, Huan Jing, Hui Liu, Lin Gao

**Affiliations:** ^1^ Department of Dermatology Xijing Hospital, Air Force Medical University Xi'an Shaanxi China

**Keywords:** laser treatment, non‐ablative fractional laser, panthenol, skin barrier repair

## Abstract

**Background:**

Facial laser procedures may result in compromised skin barrier function and associated discomfort. It is essential to address these issues to facilitate a swift recovery and enhance patient satisfaction.

**Objective:**

To evaluate the efficacy and safety of a panthenol‐enriched mask, incorporating centella asiatica extract (madecassoside) and bisabolol, in repairing the skin barrier after 1550 and 1927 nm dual‐wavelength non‐ablative fractional laser therapy.

**Methods:**

A total of 60 subjects were enrolled in this double‐blind randomized study and were divided into Control group (CG) and Mask group (MG). Both groups received standard post‐operative care, the MG receiving the Mask, while the CG received a saline dressing. Skin barrier repair was assessed using a skin physiological detection instrument, colorimetry, and a skin imaging analyzer (VISIA). The incidence of adverse reactions was also monitored.

**Results:**

The MG exhibited significantly lower erythema index and hyperpigmentation index at D3, D7, and D14, compared to the CG. The MG had significantly higher sebum content than the CG at D7 and D14. The stratum corneum moisture content in the MG was also higher than in the CG at D3, D7, and D14. Additionally, transepidermal water loss (TEWL) was significantly reduced in the MG at D3, D7, and D14. There was no significant difference in the rate of adverse reactions between the groups.

**Conclusion:**

A panthenol‐enriched Mask effectively decreased post non‐ablative laser erythema, enhanced skin hydration, and promoted skin barrier repair.

## 1 Introduction

Facial mask products are integral to post‐laser skin care regimens. As laser technology becomes more prevalent in cosmetic medicine, an increasing number of individuals seek laser treatments to address various skin concerns such as pigmentation, wrinkles, and scars. However, post‐laser skin may manifest erythema, edema, discomfort, dryness, and sensitivity, necessitating diligent care to expedite recovery and prevent complications [1]. In this context, masks, with their convenient application and immediate effects, have become popular among both professionals and patients. However, the efficacy and safety profiles of these products vary, underscoring the need for rigorous clinical evaluation. Therefore, clinical studies on facial masks for post‐laser care are of paramount importance for practical application and patient guidance.

Facial masks offer immediate hydration and soothing benefits for the skin, but also promote cellular regeneration and repair through their active ingredients [2]. The panthenol‐enriched Mask (La Roche‐Posay B5 Pro Multi action repair mask) contains various functional ingredients that support skin barrier repair, including enriched panthenol, centella asiatica extract (madecassoside), and bisabolol. Panthenol (a derivative of vitamin B5) confers antioxidant, anti‐inflammatory, and soothing properties, as well as enhancing skin hydration and barrier function [3]. Madecassoside stimulates epidermal growth factors, promoting wound healing and mitigating inflammation. Bisabolol is recognized for its anti‐inflammatory, antioxidant, antimicrobial, and wound‐healing properties, making it a valuable addition to skincare formulation [4]. Collectively, these ingredients may significantly contribute to post‐laser skin recovery.

This study aims to evaluate the clinical efficacy and safety of a panthenol‐enriched mask containing centella asiatica extract and bisabolol in post‐laser skin care.

## 2 Materials and Methods

### 2.1 Methods

The study was a single‐center, double‐blind (both investigator and subject), prospective randomized controlled trial. It comprised one treatment session and four follow‐up visits, conducted at Xijing Hospital, Air Force Medical University. The trial was approved by the Air Force Medical University Ethics Committee (Approval No. KY20232306‐F‐1) and was registered with the Chinese Clinical Trial Registry (Registration No. ChiCTR2400083929).

### 2.2 Participants

Subjects were recruited from Xijing Hospital, Air Force Medical University, Xi'an, Shaanxi, China, between May 2024 and June 2024. Inclusion criteria included aged 30–60 years, facial photoaging Global Scores for Photoaging (GSP) of 1–3, Fitzpatrick skin type II–IV, willingness to provide informed consent, absence of severe systemic disorders, no use of relevant medications within 1 month, and no use of topical antibiotics within the week prior to the study. Exclusion criteria encompassed pregnancy or planned pregnancy, lactation, a history of keloids, recent cosmetic procedures within 6 months, concurrent facial infections, active psoriasis, vitiligo, or other conditions prone to exacerbating reactions, severe systemic diseases, coagulation disorders, and significant psychiatric or psychological conditions.

### 2.3 Procedure

Sixty patients were randomly assigned to the Mask group (MG) and the Control group (CG), with 30 patients in each group, using a random number table method. The facial area was cleansed, and baseline assessments were conducted using the VISIA Skin Imaging Analyzer (7th generation, Canfield Scientific, USA), German MPA‐4 multifunctional skin tester, and Danish DSMIII colorimeter. A compounded lidocaine cream (containing 25 mg of lidocaine and 25 mg of prilocaine per gram; manufactured by Tongfang Pharmaceutical Group Co. Ltd.; Approval No. H20063466; Batch No. 230511) was uniformly applied at 40 g, covered for 40 min for surface anesthesia. The area was then disinfected with 75% alcohol. Non‐ablative fractional laser treatment was conducted using a dual‐wavelength 1550 and 1927 nm system (1550 nm at 20 J/cm^2^ for 4 passes; 1927 nm at 10 J/cm^2^ for four passes). A cold spray was applied for 20 min during and immediately post‐treatment, followed by the dressing application for 15–20 min. The MG applied the La Roche‐Posay B5 Pro Multi action repair mask [L'Oréal (China) Ltd.], while the CG applied a saline dressing. Post‐treatment care included avoiding wetting the face with water for 1 day. After 1 day, both groups used the respective dressings once daily for 14 days, with each application lasting 15–20 min. After dressing removal, the face was cleaned with water, and a prebiotic and panthenol containing repairing balm (La Roche‐Posay Cicaplast balm B5+) was applied for moisturizing in both groups. Participants were instructed to use physical sun protection and SPF > 30 sunscreen.

The study lasted for 14 days, with visits on the treatment day (D0), day 1 (D1, 15–20 min post‐application of mask), day 3 (D3), day 7 (D7), and day 14 (D14).

### 2.4 Assessment

Instrumental Assessment: Skin sebum content, stratum corneum hydration (SCH), and transepidermal water loss (TEWL) were measured using the German MPA‐4 multifunctional skin tester at each visit. The erythema index (EI) and melanin index (MI) were assessed using the Danish DSMIII colorimeter.

Clinical Assessment: Clinical photographs were captured at each visit using the VISIA Skin Imaging Analyzer (7th generation, Canfield Scientific, USA). Postoperative adverse reactions were documented.

### 2.5 Statistical Analysis

Data analysis was performed using SPSS version 22.0. Continuous variables were presented as mean ± SD. Between‐group comparisons were conducted using analysis of variance (ANOVA), with pairwise comparisons performed using LSD‐*t* tests and intergroup comparisons using independent sample t tests. Categorical data were expressed as percentages (%), and group comparisons were conducted using *χ* ^2^ or adjusted *χ* ^2^ tests. A *p* < 0.05 was considered statistically significant.

## 3 Results

### 3.1 Participant Demographics

A total of 60 participants with photoaging were recruited in the study, including 57 females and 3 males. The age range was 32–54 years, with an average age of 39.0 ± 6.2 years. All participants successfully completed the treatment and follow‐up phases. There were no statistically significant differences between the two groups in terms of age, sex, skin type, or Global Scores for Photoaging (GSP) scores (*p* > 0.05) (Table 1).

**TABLE 1:** Demographic characteristics of participants in two different assessment groups.

	MG	CG	*t*	*p*
Number	30	30		
Age (mean ± SD)	38.53 ± 6.88	39.5 ± 5.61	−0.596	0.553
Gender				
Male	2	1	0.351	0.554
Female	28	29		
Phototype (*n*, %)				
II	9	5	2.058	0.357
III	19	24		
IV	2	1		
GSP score				
1	3	2	1.156	0.561
2	17	14		
3	10	14		

### 3.2 Efficacy Evaluation

#### 3.2.1 Instrumental Assessment

The erythema index of the lesions in the MG was significantly lower than in the CG at D3, D7, and D14 (D3: CG: 19.15 ± 4.90, MG: 16.25 ± 4.75, *p* < 0.05; D7: CG: 13.48 ± 4.04, MG: 11.47 ± 2.17, *p* < 0.05; D14: CG: 13.27 ± 2.97, MG: 11.02 ± 1.93, *p* < 0.01) (Table 2).

**TABLE 2:** Comparison of Erythema Index (EI) and Melanin Index (MI) at Lesion Area Between Groups.

Group	Sample size	EI					MI				
		D0	D1	D3	D7	D14	D0	D1	D3	D7	D14
MG	30	14.15 ± 5.11	17.19 ± 3.51	16.25 ± 4.75	11.47 ± 2.17	11.02 ± 1.93	37.41 ± 2.28	38.81 ± 2.64	38.32 ± 2.98	36.27 ± 2.25	35.23 ± 2.44
CG	30	13.94 ± 4.05	16.82 ± 2.71	19.15 ± 4.90	13.48 ± 4.04	13.27 ± 2.97	37.09 ± 2.30	39.04 ± 2.53	40.09 ± 2.99	38.18 ± 2.15	36.96 ± 1.85
*t*		−0.170	0.185	2.337	2.400	3.474	−0.5500	0.118	2.295	3.362	3.101
*p*		0.865	0.668	0.023	0.020	0.001	0.584	0.732	0.025	0.001	0.003

Similarly, the melanin index of the lesions in the MG was significantly lower than in the CG at D3, D7, and D14 (D3: CG: 40.09 ± 2.99, MG: 38.32 ± 2.98, *p* < 0.05; D7: CG: 38.18 ± 2.15, MG: 36.27 ± 2.25, *p* < 0.01; D14: CG: 36.96 ± 1.85, MG: 35.23 ± 2.44, *p* < 0.01) (Table 2).

Compared to the CG, the MG demonstrated a significantly greater increase in facial sebum content after 7 and 14 days with the panthenol‐containing dressings (D7: CG: 41.85 ± 16.03, MG: 50.84 ± 13.91, *p* < 0.01; D14: CG: 45.79 ± 12.21, MG: 50.87 ± 10.55, *p* < 0.05). No significant difference in sebum content was noted between the two groups on D3 (*p* = 0.239) (Table 3).

**TABLE 3:** Comparison of skin barrier function between groups.

Group	Sample size	Sebum content (μg/cm^2^)				Stratum corneum hydration (%)				TEWL [g/(h·m^2^)]			
		D0	D3	D7	D14	D0	D3	D7	D14	D0	D3	D7	D14
Observation group	30	41.01 ± 15.7	40.99 ± 16.72	50.84 ± 13.91	50.87 ± 10.55	46.86 ± 10.22	37.57 ± 12.80	37.22 ± 7.77	51.55 ± 8.81	25.02 ± 6.80	18.37 ± 7.54	12.63 ± 4.54	11.49 ± 5.46
Control group	30	41.43 ± 13.57	35.72 ± 13.74	41.85 ± 16.03	45.79 ± 12.21	43.73 ± 11.57	30.95 ± 11.11	32.99 ± 11.48	44.76 ± 9.62	25.73 ± 5.62	23.64 ± 9.33	20.5 ± 9.6	16.11 ± 5.57
*t*		0.111	−1.189	−0.306	−2.302	−1.11	−2.138	−2.28	−2.848	0.498	−2.349	−3.958	−3.023
*p*		0.912	0.239	0.003	0.024	0.271	0.037	0.026	0.006	0.659	0.019	0	0.002

Stratum corneum hydration increased in both groups following treatment, with the MG showing significantly higher levels compared to the CG at D3, D7, and D14 (D3: CG: 30.95 ± 11.11, MG: 37.57 ± 12.80, *p* < 0.05; D7: CG: 32.99 ± 11.48, MG: 37.22 ± 7.77, *p* < 0.05; D14: CG: 44.76 ± 9.62, MG: 51.55 ± 8.81, *p* < 0.01) (Table 3).

Transepidermal water loss (TEWL) was significantly reduced in the MG compared to the control group at D3, D7, and D14 (D3: CG: 23.64 ± 9.33, MG: 18.37 ± 7.54, *p* < 0.05; D7: CG: 20.50 ± 9.60, MG: 12.63 ± 4.54, *p* < 0.001; D14: CG: 16.11 ± 5.57, MG: 11.49 ± 5.46, *p* < 0.01) (Table 3).

#### 3.2.2 Clinical Evaluation

**FIGURE 1:**
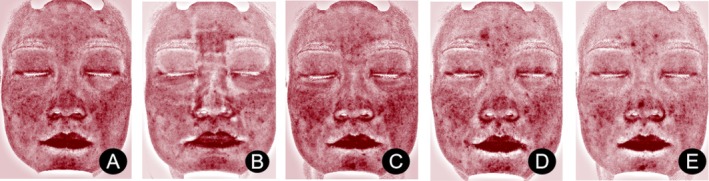
Changes in the red area of the treatment zone in the MG on D0 (A), D1 (B, 15–20 min post‐application of mask), D3 (C), D7 (D), and D14 (E).

**FIGURE 2:**
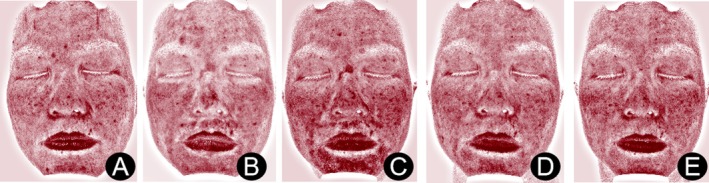
Changes in the red area of the treatment zone in the CG on D0 (A), D1 (B), D3 (C), D7 (D), and D14 (E).

The MG exhibited more pronounced reductions in facial erythema and swelling following treatment, aligning with the lower erythema and melanin indexes observed at D3, D7, and D14 (Figures 1 and 2).

### 3.3 Safety

In the MG, three cases (10%) of adverse reactions were reported. All reactions involved unilateral facial flushing, which resolved with increased moisturization within 3–5 days. None of these were considered related to the mask product, and all three patients continued the treatment regimen without discontinuation. In the CG, one patient experienced facial erythema (3.3%). The difference in the incidence of adverse reactions between the two groups was not statistically significant (*χ* ^2^ = 1.071, *p* = 0.301).

### 3.4 Typical Cases

**FIGURE 3:**
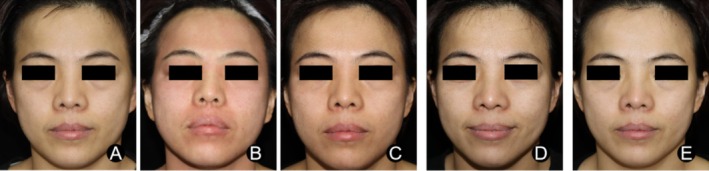
Typical case (MG) (A) D0; (B) D1; (C) D3; (D) D7; (E) D14, showing no significant erythema or dryness on the face.

*Case 1 (MG)*: A 34‐year‐old female underwent 1550 and 1927 nm non‐ablative fractional laser treatment for facial photoaging. Postoperatively, the patient experienced facial erythema, swelling, and dryness. After the application of La Roche‐Posay Mask Pro in addition to standard care, significant improvements in erythema and swelling were observed by D14 (Figure 3).

**FIGURE 4:**
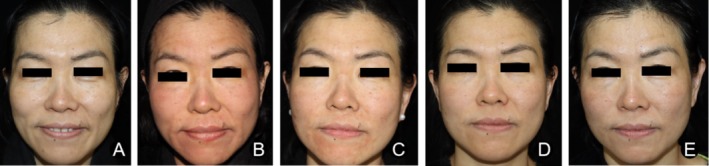
Typical case (CG) (A) D0; (B) D1; (C) D3; (D) D7; (E) D14, showing persistent erythema, swelling, and dryness on the cheeks on D14.

*Case 2 (CG)*: A 42‐year‐old female received the same laser treatment and standard postoperative care. The improvement in facial erythema and dryness improved more slower, with persistent erythema on the cheeks on D14 (Figure 4).

## 4 Discussion

Non‐ablative fractional laser treatment is a valuable approach for facial phototherapy, offering skin remodeling without epidermis removal. It has shown promising results in treating conditions such as acne and photoaging. The 1550 nm wavelength targets deeper dermal structures, addressing mild to moderate skin laxity, scarring, and photoaging [5, 6]. In contrast, the 1927 nm wavelength non‐ablative fractional laser affects the superficial dermis, enhancing skin texture and reducing pigmentation [7, 8]. Combined treatment with these lasers is also applied in the management of facial photoaging [9]. However, non‐ablative fractional lasers may cause thermal and mechanical damage to the tissue, potentially compromising skin barrier function, thus increasing the focus on postoperative erythema, pain, and post‐inflammatory pigmentation. Consequently, postoperative repair interventions have become a significant area of interest following laser procedures.

Vitamin B5, or pantothenic acid, is a water‐soluble vitamin essential for the biosynthesis of coenzyme A [10]. Pantothenic acid's precursor, D‐panthenol, interacts with the extracellular lipid and protein layers of epidermal keratinocytes, reducing moisture loss by maintaining or increasing molecular fluidity, thereby exhibiting moisturizing and barrier‐improving properties. It is commonly used in skincare and haircare products [11]. Additionally, panthenol‐containing products are utilized in the treatment and prevention of xerosis, pruritus, and skin irritations such as atopic dermatitis and diaper dermatitis [12]. In vitro studies have shown that panthenol can upregulate genes associated with wound healing and play a significant role in wound repair, scar management, and hair regrowth [12]. Prospective clinical studies also suggest that topical panthenol can promote the rapid re‐epithelialization of damaged skin and restoration of skin barrier function, thus facilitating wound healing. Therefore, topical panthenol for superficial skin injuries may be a rational choice [13].

In addition to panthenol, the panthenol‐enriched mask contains several effective components, including madecassoside (centella asiatica extract) and bisabolol. Madecassoside is known for its anti‐aging, skin hydration, collagen synthesis promotion, ultraviolet protection, and scar treatment properties [13]. It also facilitates the repair of skin lesions following laser procedures and reduces the occurrence of postoperative adverse reactions [14]. Bisabolol, an unsaturated monoterpene alcohol, exerts anti‐inflammatory effects by reducing TNF‐α, IL‐1β, IL‐6, iNOS, and COX‐2, while also possessing antioxidant properties [14].

Previous studies have shown that the panthenol‐enriched mask improves stratum corneum hydration, sebum production, and transepidermal water loss in patients with dry sensitive skin, oily sensitive skin, and oily acne‐prone skin [15]. It also alleviates erythema, edema, post‐inflammatory erythema, and pigmentation, with good tolerance [1]. In this study, the panthenol‐enriched mask was applied postoperatively following 1550 and 1927 nm dual‐wavelength non‐ablative fractional laser treatment. Results indicated that patients using the panthenol‐enriched mask experienced a significant increase in sebum content and stratum corneum hydration compared to the CG. This suggests that the panthenol‐enriched mask significantly improves postoperative skin hydration, enhances skin barrier function after laser treatment, and promotes the recovery of postoperative wounds. Additionally, erythema and melanin indexes on D3, D7, and D14 were significantly reduced in the MG compared to the CG, indicating that the panthenol‐enriched mask helps decrease the severity of postoperative erythema and pigmentation.

The findings of this study align with and extend previous work investigating the efficacy of panthenol‐based formulations in enhancing skin recovery post‐laser treatment. Prior studies have demonstrated that topical panthenol significantly improves stratum corneum hydration and accelerates epidermal barrier restoration. For instance, Zhang et al. [15] reported that a panthenol‐enriched mask significantly improved hydration and reduced transepidermal water loss in individuals with impaired skin barrier subtypes. Similarly, Lueangarun et al. [13] conducted a split‐face trial comparing a moisturizer containing 5% panthenol, madecassoside, and copper‐zinc‐manganese to topical corticosteroids following ablative laser treatment and found superior tolerance and equivalent efficacy in reducing downtime and erythema. Our study corroborates these findings in the context of dual‐wavelength non‐ablative fractional laser therapy, showing enhanced improvements in erythema, melanin index, sebum content, and TEWL in the panthenol mask group compared with standard saline dressing. The benefits of the tested mask are likely multifactorial. Panthenol serves as a humectant and skin‐conditioning agent, promoting hydration and barrier repair [3]. Madecassoside, derived from *Centella asiatica* , has been shown to upregulate epidermal growth factors, stimulate collagen synthesis, and reduce inflammation, contributing to dermal remodeling and wound healing. Bisabolol, a natural sesquiterpene alcohol, exerts anti‐inflammatory, antioxidant, and antimicrobial effects that may mitigate laser‐induced irritation and support tissue regeneration [4]. Collectively, these ingredients appear to act synergistically, accelerating re‐epithelialization while minimizing post‐inflammatory erythema and pigmentation. Compared with previous formulations used post‐laser, the mask evaluated in this study offers a convenient, well‐tolerated, and non‐pharmacologic option that integrates barrier‐supportive and anti‐inflammatory properties into a single‐step application. These attributes may enhance patient adherence and satisfaction in real‐world clinical practice.

One limitation of this study is the potential confounding effect of occlusion. The intervention group received a panthenol‐enriched occlusive mask, while the control group used a non‐occlusive saline compress. Although the mask contains bioactive ingredients with documented benefits for skin repair, the occlusive nature of the dressing itself may have independently contributed to improvements in hydration and barrier function by reducing transepidermal water loss. Therefore, we cannot entirely exclude the possibility that part of the observed efficacy may be attributable to the physical properties of the mask rather than its active constituents alone. Future studies employing a comparator with matched occlusive properties but lacking active ingredients would be valuable to further isolate the pharmacologic effects. Another limitation of this study is that it was conducted following non‐ablative fractional laser treatment, which causes less extensive epidermal disruption than ablative procedures. Future studies are warranted to evaluate the mask's efficacy in the context of more profound barrier damage induced by ablative laser treatments.

In summary, the panthenol‐enriched mask is beneficial for reducing pigmentation and erythema in patients following non‐ablative fractional laser treatment. It significantly promotes the restoration of skin barrier function and helps in postoperative skin hydration, demonstrating considerable clinical value.

## Author Contributions

All authors contributed to the study conceptualization and design. Meiyan Gao was responsible for material preparation, data collection, and analysis. The initial draft of the manuscript was authored by Meiyan Gao, with subsequent revisions and comments provided by all authors. The final manuscript has been read and approved by all authors.

## Ethics Statement

The study was conducted with the approval of the Air Force Medical University Ethics Committee (approval no. KY20232306‐F‐1). Informed consent was secured from all subjects prior to their inclusion in the study.

## Consent

All participants provided informed consent, which included the use of personal data, including any personal details or images.

*Photo consent*: Written informed consent was obtained from the patient/subject for the publication of their photograph. The editorial board of this journal may request to review copies of the informed consent forms.

*Consent for publication*: The manuscript is submitted with the full approval of all listed authors.

## Conflicts of Interest

The authors declare no conflicts of interest.

## Data Availability

The data that support the findings of this study are available from the corresponding author upon reasonable request.
